# Jugular Paraganglioma Presenting As Isolated Hypoglossal Nerve Palsy: An Anatomical Enigma

**DOI:** 10.7759/cureus.100337

**Published:** 2025-12-29

**Authors:** Manir Chujfi, Luis D Marquez-Farias, Ildefonso Rodriguez-Leyva

**Affiliations:** 1 Neurology, Hospital Central Dr. Ignacio Morones Prieto, San Luis Potosi, MEX; 2 Neurology, "Dr. Ignacio Morones Prieto" Regional High Specialty Hospital, San Luis Potosi, MEX; 3 Neurology, Autonomous University of San Luis Potosi, San Luis Potosi, MEX

**Keywords:** collet-sicard syndrome, cranial neuropathies, hypoglossal nerve palsy, jugular foramen, jugular paraganglioma, lower cranial nerves

## Abstract

Hypoglossal nerve palsy is most commonly associated with trauma, tumors, or vascular insults affecting the skull base, but isolated palsy due to jugular paraganglioma is extremely rare. We present the case of a woman in her 40s with progressive dysarthria and isolated left hypoglossal nerve palsy. MRI revealed a vascular lesion localized in the left jugular foramen with extension into the hypoglossal canal, sparing the adjacent lower cranial nerves. The absence of involvement of cranial nerves IX, X, and XI ruled out a complete Collet-Sicard syndrome. Imaging characteristics and anatomical correlation were clearly consistent with a jugular paraganglioma. Given the slow progression, high morbidity associated with surgical resection, and the patient's clinical stability, a deep discussion by a multidisciplinary team recommended non-invasive management. Although stereotactic radiosurgery and succinate dehydrogenase (SDH) mutation testing were offered, the patient declined both and opted for clinical surveillance. This case underscores the importance of considering jugular paragangliomas in the differential diagnosis of isolated cranial neuropathies, especially when typical syndromic presentations are absent. The selective involvement of the hypoglossal nerve without apparent and concurrent dysfunction of adjacent lower cranial nerves represents a rare anatomical enigma, likely explained by the spatial relationships and embryological separation between the hypoglossal and jugular foramina. High-resolution imaging and collaborative multidisciplinary decision-making remain essential in tailoring patient-centered treatment strategies for skull base tumors.

## Introduction

Paragangliomas are rare neuroendocrine tumors arising from neural crest-derived paraganglia associated with the autonomic nervous system [1,2]. Head and neck paragangliomas account for 65-70% of all paragangliomas, with an estimated incidence of 0.3-1 per 100,000 persons [3]. Jugular paragangliomas (glomus jugulare tumors) are slow-growing, hypervascular neoplasms arising within the jugular foramen or jugular bulb adventitia [1,4]. They represent the most common tumor of the jugular fossa and occur with an annual incidence of 1 per 1.3 million people, predominantly in women (female-to-male ratio 3-6:1) aged 40-70 years [4]. Approximately 10-40% have a hereditary component, most commonly involving germline mutations in succinate dehydrogenase (SDH) genes, which encode mitochondrial enzymes involved in cellular respiration [1,3].

The jugular foramen, located between the occipital and temporal bones at the skull base, transmits cranial nerves (CNs) IX (glossopharyngeal), X (vagus), and XI (accessory), along with the sigmoid sinus continuing as the internal jugular vein [5]. The hypoglossal canal, transmitting CN XII, lies medially and anteriorly to the jugular foramen, separated by the jugular tubercle [5,6]. This anatomical arrangement typically results in jugular paragangliomas presenting with pulsatile tinnitus, progressive hearing loss, or multiple lower cranial nerve deficits, particularly CN IX-XI, constituting Collet-Sicard syndrome (combined palsy of CNs IX, X, XI, and XII) when all four nerves are involved [1,2].

Isolated hypoglossal nerve palsy (CN XII) is uncommon. Due to CN XII's anatomical proximity to other lower CNs and medullary structures, palsy typically presents with concurrent involvement of adjacent nerves [7]. A review of 100 cases of hypoglossal nerve palsy revealed that tumors, predominantly malignant, accounted for nearly half of the cases, with only 15% achieving complete recovery [7]. Isolated hypoglossal nerve palsy as the sole manifestation of a jugular paraganglioma is exceedingly rare and not well documented in the literature [8]. Neuroimaging, particularly MRI with gadolinium, plays a crucial role, as these tumors demonstrate characteristic intense enhancement and the classic "salt-and-pepper" appearance on T1- and T2-weighted images (due to flow voids from tumor hypervascularity) [9].

This case report adds to the limited literature by documenting isolated CN XII involvement - a presentation not previously well characterized for jugular paragangliomas. We demonstrate that paragangliomas can selectively affect the hypoglossal nerve without the typical syndromic involvement of CN IX-XI (Collet-Sicard syndrome), likely through extrinsic compression rather than direct canal invasion. This atypical presentation has important diagnostic implications, as it broadens the differential diagnosis of isolated tongue paresis and underscores the need for vascular skull-base imaging even when classical signs are absent.

## Case presentation

A woman in her 40s presented to our outpatient neurology clinic with a three-month history of progressive slurred speech and difficulty articulating words. The patient reported noticing tongue weakness approximately two weeks before developing noticeable speech difficulties, with gradual progression over the subsequent three-month period prior to presentation. There were no associated symptoms of dysphagia, hearing loss, hoarseness, or neck pain at initial presentation. Notably, the patient also reported paroxysmal episodes of pulsatile headache, palpitations, intermittent arterial hypertension, and excessive sweating (diaphoresis). There was no history of trauma, recent infections, or constitutional symptoms such as weight loss or night sweats.

Her past medical history was unremarkable, and she reported no use of medications. There was no known family history of neurological or neoplastic diseases. She lived in an urban region of central Latin America and worked in administrative duties, without known occupational exposure to toxins.

Neurological examination revealed isolated left-sided tongue deviation with visible hemiatrophy and fasciculations, consistent with a lower motor neuron lesion of the hypoglossal nerve. Speech examination revealed moderate dysarthria with preserved intelligibility at approximately 85-90%, primarily affecting lingual sounds. Formal swallowing evaluation was normal with no evidence of dysphagia. There were no signs of involvement of CNs IX, X, or XI. Motor and sensory examinations of the limbs were normal, as were cerebellar and reflex assessments. Table 1 shows the timeline of the presentation.

**Table 1:** Clinical timeline.

Time point	Clinical events
Week 0	Patient noticed left tongue weakness.
Week 2	Progressive dysarthria developed.
Week 12	Presentation to the neurology clinic. Isolated cranial nerve XII palsy confirmed, with no involvement of cranial nerves IX-XI and no autonomic symptoms reported.
Weeks 12-13	Diagnostic workup: magnetic resonance imaging revealed a 2.1-cm jugular foramen lesion; computed tomography showed bone erosion with an intact hypoglossal canal; laboratory testing demonstrated elevated metanephrine levels consistent with a functional tumor; audiometry was normal.
Week 14	Multidisciplinary evaluation. Conservative management was selected, and radiation therapy was planned.

**Figure 1:**
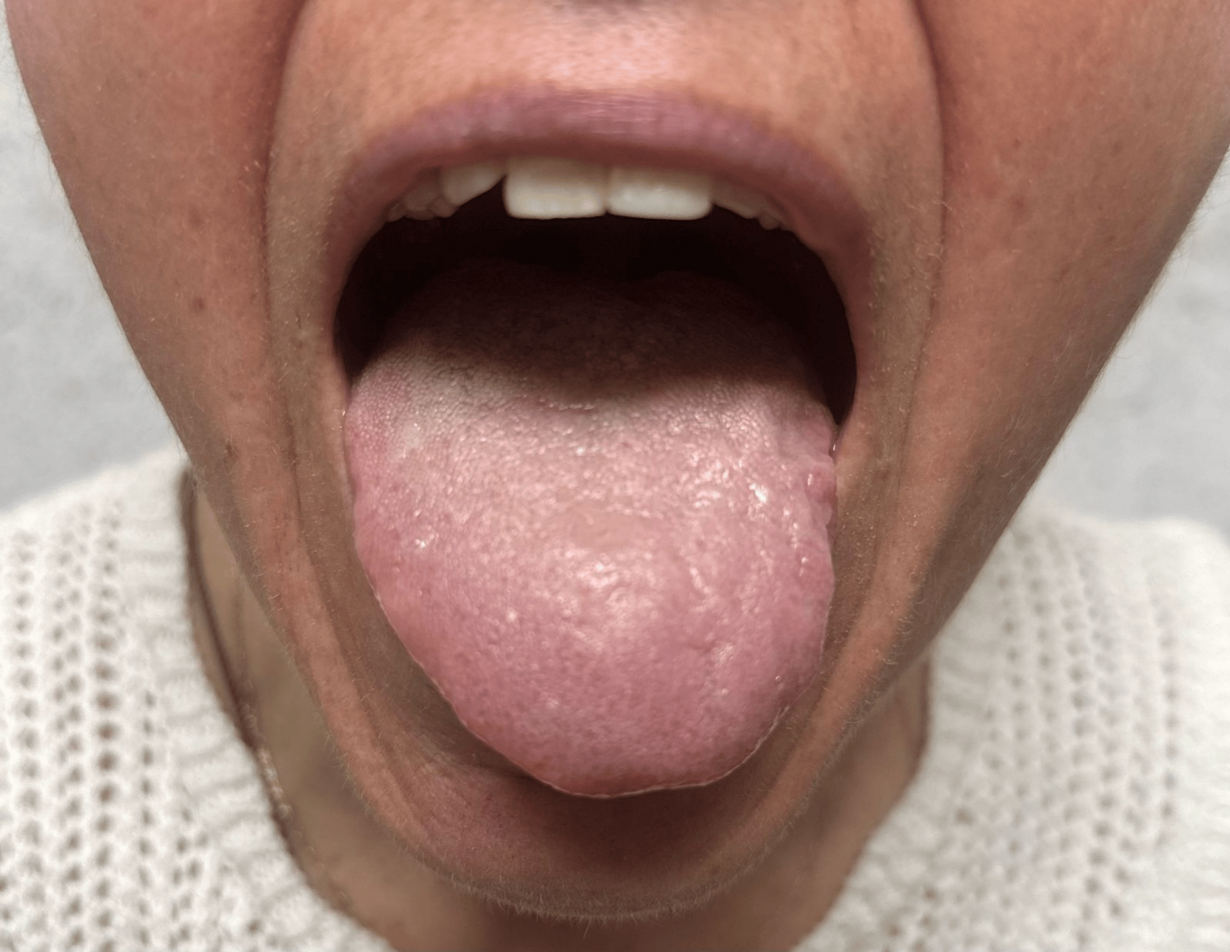
Clinical photograph of the patient with the mouth open, showing tongue deviation to the left and fasciculations of the left hemi-tongue. Written informed consent for publication was obtained from the patient.

A clinical photograph (Figure 1) was obtained showing the tongue at rest with apparent deviation to the left and visible fasciculations on the same side, supporting the diagnosis of isolated hypoglossal nerve palsy.

Additionally, a video was recorded during the clinical examination (Video 1), in which the patient is instructed to protrude her tongue and move it sequentially to the right, left, and upward. The tongue shows marked deviation to the left with impaired lateral mobility and prominent resting fasciculations on the left side.

**Video 1:** The patient is instructed to move her tongue to the right, left, and upward. Marked deviation to the left and resting fasciculations of the left side are demonstrated. Written informed consent for publication was obtained from the patient.

**Figure 2:**
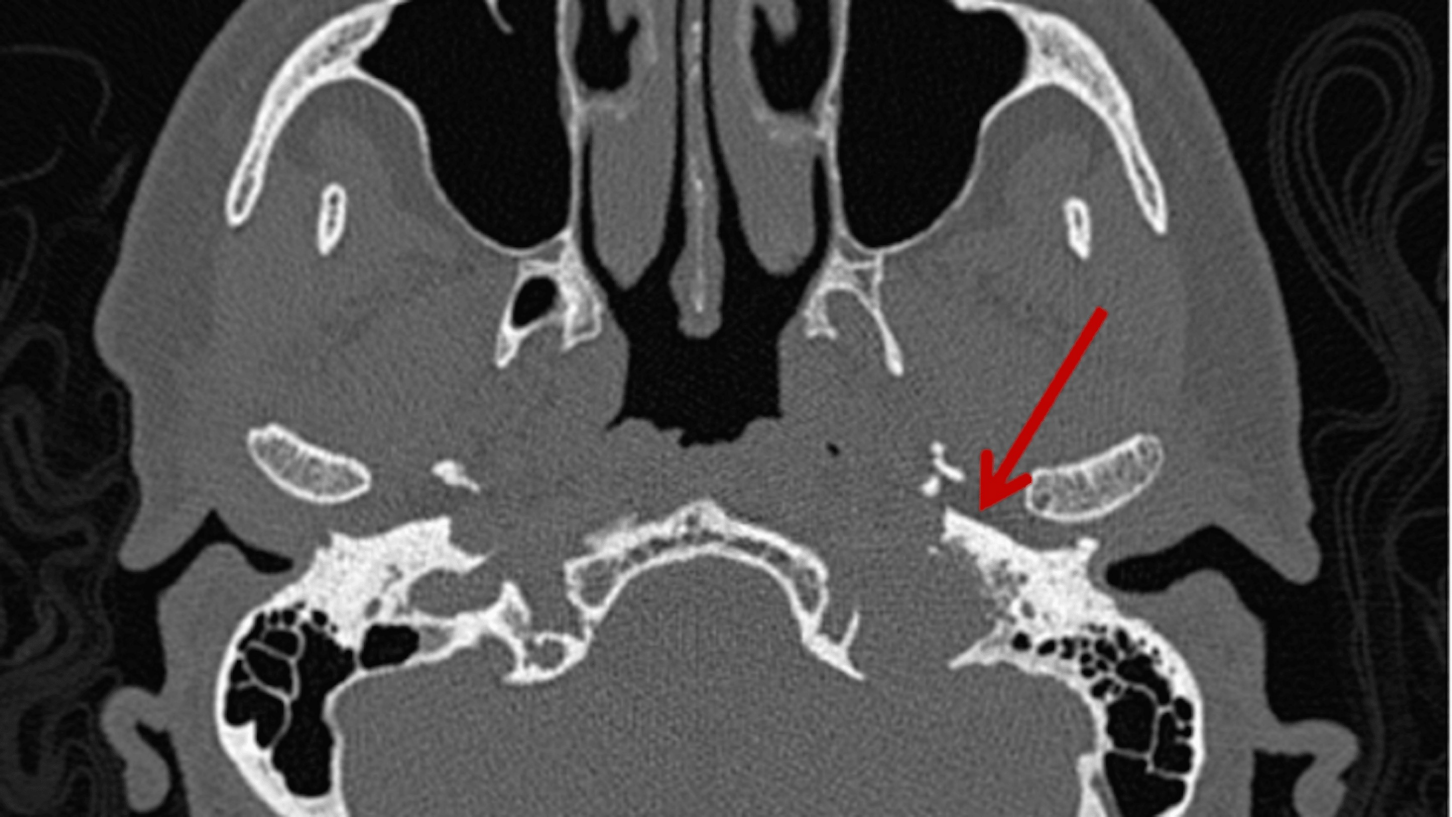
Axial non-contrast-enhanced computed tomography scan of the skull base at the level of the jugular foramen. Erosion of the left jugular foramen is evident (red arrow), with mild asymmetry of the jugular bulbs, suggesting an expansive lesion in this region consistent with a jugular paraganglioma.

**Figure 3:**
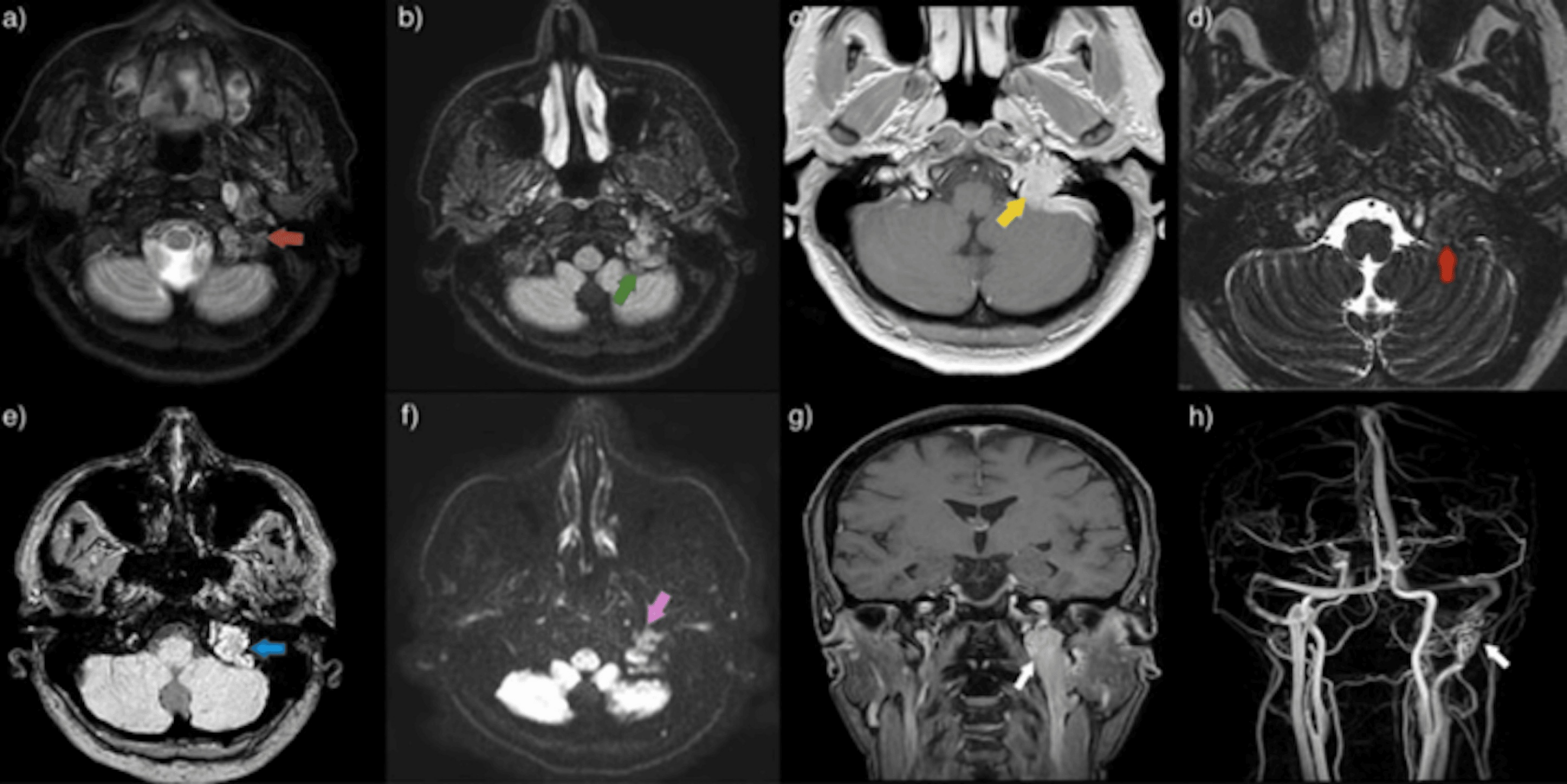
Mosaic of axial and angiographic magnetic resonance imaging sequences. Magnetic resonance imaging sequences, with and without gadolinium contrast, demonstrate a space-occupying lesion involving the left jugular foramen with bone erosion, prominent vascular structures arising from the external carotid artery, and venous drainage into an enlarged left internal jugular vein, consistent with a jugular paraganglioma (glomus jugulare). The lesion measures approximately 1.7 × 2.4 × 8.4 cm (anteroposterior × transverse × craniocaudal). (a) Axial T2-weighted fast spin-echo image showing an isointense lesion relative to cerebellar tissue at the level of the jugular bulb (orange arrow). (b) Axial T2-weighted FLAIR image showing the lesion at the left jugular bulb (green arrow). (c) Post-contrast T1-weighted gradient-echo image demonstrating avid enhancement of the lesion (yellow arrow). (d) Heavily T2-weighted axial image highlighting the lesion at the jugular bulb (red arrow) with a characteristic “salt-and-pepper” appearance due to flow voids from tumor hypervascularity. (e) Axial susceptibility-weighted (VenBOLD) image confirming the hypervascular lesion (light blue arrow). (f) Axial diffusion-weighted image revealing signal isointensity of the left jugular glomus (pink arrow). (g) Coronal post-contrast T1-weighted spin-echo image showing the lesion occupying the left jugular bulb (white arrow). (h) Three-dimensional phase-contrast angiographic reconstruction demonstrating hypervascularity of the lesion with arterial supply from branches of the external carotid artery (white arrow). FLAIR: fluid-attenuated inversion recovery

Given the isolated cranial nerve deficit and the presence of autonomic symptoms, a plasma catecholamine profile was obtained, including free normetanephrines, plasma metanephrines, and total metanephrines, all of which were elevated. Results revealed elevated metanephrine levels, including plasma free normetanephrines of 292 pg/mL (reference range <148 pg/mL), plasma free metanephrines of 63 pg/mL (reference range <57 pg/mL), and total metanephrines of 355 pg/mL (reference range <205 pg/mL). All three values exceeded normal limits, confirming a biochemically active (functional) paraganglioma. Additionally, an initial non-contrast computed tomography (CT) scan of the head was performed, revealing widening and erosion of the left jugular foramen, along with mild asymmetry of the jugular bulbs (Figure 2). These findings prompted further evaluation with contrast-enhanced magnetic resonance imaging (MRI), which demonstrated a well-defined, hypervascular lesion centered on the left jugular foramen with avid gadolinium enhancement and no extension into the hypoglossal canal or adjacent brain structures. These findings are consistent with a jugular paraganglioma (glomus jugulare) (Figure 3). No additional intracranial abnormalities were identified.

The patient was referred for further evaluation by a skull-base multidisciplinary team, including neurosurgery and radiation oncology. Additional imaging ruled out multifocal disease, while biochemical screening confirmed elevated catecholamine secretion, establishing the diagnosis of a functional paraganglioma. Table 2 shows the features of the suspicion of the diagnosis.

**Table 2:** Key features for the diagnosis of jugular paraganglioma.

Category	Features
Key features that should prompt suspicion	Isolated progressive cranial neuropathy (especially cranial nerve XII alone); unilateral tongue deviation, atrophy, and fasciculations with no pulsatile tinnitus, no other cranial nerve involvement, and normal hearing; absence of upper motor neuron signs; no alternative explanation such as trauma, stroke, or demyelination
Consider even if	Pulsatile tinnitus is absent; multiple cranial nerves are not involved; hearing is normal; no autonomic symptoms (functional tumors can be asymptomatic)
Essential workup	Skull base magnetic resonance imaging with contrast; computed tomography for bone detail; plasma metanephrine testing to identify functional tumors

## Discussion

Paragangliomas of the jugular foramen are highly vascular tumors that typically involve the lower CNs, most frequently IX, X, and XI, due to their correlated anatomical trajectory within the jugular foramen [10]. In contrast, isolated involvement of the hypoglossal nerve (XII) is exceedingly rare, given its independent course through the hypoglossal canal, which lies medial and anterior to the jugular foramen [11]. In this case, despite imaging findings consistent with a glomus jugulare, the patient presented solely with left-sided tongue deviation and fasciculations, without involvement of other CNs. This presentation is infrequent and diagnostically intriguing.

The hypoglossal nerve (CN XII) originates as several rootlets from the medulla oblongata between the pyramid and the olive, traversing the premedullary cistern before entering the hypoglossal canal, a bony tunnel located superior and medial to the jugular foramen [11,12]. Upon exiting the canal, it courses anterolaterally toward the carotid space. Anatomically, CN XII is independent from the lower CNs (IX-XI), which exit through the jugular foramen in proximity, but via separate dural and osseous compartments [12]. We hypothesize that the isolated hypoglossal palsy may have resulted from extrinsic compression of the nerve near its exit from the canal or at its cisternal segment, rather than direct invasion of the canal itself. This atypical pattern highlights the potential for selective CN XII involvement even when the primary lesion does not anatomically traverse the hypoglossal canal.

In this patient, MRI revealed asymmetry of the jugular bulbs with enlargement and a well-defined, contrast-enhancing vascular lesion centered on the left jugular foramen, suggestive of a jugular paraganglioma. The lesion extended toward the hypoglossal canal, though no direct invasion of the canal was observed on MRI. However, subsequent non-contrast CT imaging demonstrated clear, marked bony erosion of the left jugular foramen, consistent with chronic remodeling secondary to a hypervascular mass. Notably, no definitive erosion of the hypoglossal canal itself was seen.

Despite this location, no clinical or radiological evidence of dysfunction in CNs IX, X, or XI was found, nor was there definitive extension of the mass into the hypoglossal canal on MRI or CT. Thus, we hypothesize that the isolated hypoglossal palsy may have resulted from extrinsic compression of the nerve near its exit from the canal or at its cisternal segment, rather than direct invasion of the canal itself. This atypical pattern highlights the potential for selective CN XII involvement even when the primary lesion does not anatomically traverse the hypoglossal canal. The exact mechanism by which the tumor selectively affects CN XII while sparing adjacent structures remains incompletely understood and warrants further investigation with advanced imaging techniques or, if surgical resection is eventually pursued, direct anatomical observation.

Neuroimaging was instrumental in establishing the diagnosis [13]. High-resolution MRI with and without contrast, along with MR angiography, revealed a well-defined, hypervascular mass centered at the left jugular foramen, exhibiting avid gadolinium enhancement and prominent flow voids. The lesion's signal characteristics, including T2 isointensity and absence of diffusion restriction or hemorrhage, strongly suggested a paraganglioma. The tumor's medial extension suggested possible proximity to the hypoglossal canal, although the initial MRI suggested no definitive hypoglossal canal invasion. CT imaging clearly demonstrated marked osseous erosion of the left jugular foramen, consistent with chronic pressure remodeling. Additionally, plasma catecholamine testing revealed elevated levels of normetanephrines and total metanephrines, supporting the diagnosis of a functionally active paraganglioma rather than a non-functional lesion [14].

The differential diagnosis of jugular foramen lesions is broad and includes schwannomas, meningiomas, metastases, chordomas, lymphomas, and inflammatory or infectious processes (Table 3). However, the imaging characteristics in this case were highly specific for jugular paraganglioma. The combination of intense enhancement with salt-and-pepper appearance on T1- and T2-weighted images, prominent flow voids representing tumor hypervascularity, and permeative bone erosion of the jugular foramen without hyperostosis strongly favored paraganglioma over other entities. Schwannomas typically demonstrate smooth bone remodeling rather than permeative destruction and lack the prominent vascularity seen in this case. Meningiomas characteristically show hyperostosis and a dural tail sign, neither of which was present. Metastatic lesions would be expected to show more aggressive, rapid bone destruction with irregular margins and often multiple sites of involvement. The absence of systemic symptoms, normal inflammatory markers, and typical imaging features effectively excluded infectious and inflammatory etiologies [15]. However, these entities often lack the intense vascularity and characteristic imaging features seen in this case. Importantly, the absence of systemic symptoms or diffuse contrast enhancement made infectious and inflammatory causes less likely [16]. For comparative purposes, Table 3 provides a differential diagnosis based on diagnostic imaging, highlighting the key radiological features that help distinguish paragangliomas from other skull base lesions.

**Table 3:** Differential diagnosis of jugular foramen lesions. CN: cranial nerve; CPA: cerebellopontine angle; CSF: cerebrospinal fluid; CT: computed tomography; DM: diabetes mellitus; F:M: female to male ratio; IgG4-RD: IgG4-related disease; MRI: magnetic resonance imaging; STIR: short tau inversion recovery; ESR: erythrocyte sedimentation rate; IgG4-RD: immunoglobulin G4-related disease

Diagnosis	MRI characteristics	Clinical features	CT findings	Key distinguishing features
Jugular paraganglioma (glomus jugulare)	Intense enhancement	Pulsatile tinnitus (most common)	Permeative bone erosion	Highly vascular with prominent flow voids
	"Salt-and-pepper" appearance on T1/T2	Lower CN palsies (IX-XI typical, XII rare)	"Moth-eaten" pattern	Salt-and-pepper appearance pathognomonic
	Multiple flow voids	Conductive hearing loss	Jugular foramen enlargement	Strong arterial enhancement
	Isointense on T1, hyperintense on T2	Rarely functional (catecholamine secretion <5%)	No bony matrix	External carotid artery supply
	No diffusion restriction	F:M = 4-6:1 Age: 40-70 years	Hypervascular on angiography	-
Schwannoma (CN IX, X, or XI)	Moderate-intense enhancement	Progressive CN deficit (usually single nerve)	Smooth bone remodeling	Lacks prominent vascularity
	Cystic degeneration common	No pulsatile tinnitus	Expanded but intact margins	Smooth, expansile growth pattern
	T1: Iso- to hypointense	Hearing loss if extends to CPA	Jugular foramen enlargement	Cystic changes common
	T2: Hyperintense	No functional symptoms. Age: 30-60 years	No permeative destruction	No salt-and-pepper
	Target sign (central hypointensity)	Slow growing	Well-defined borders	Less enhancement than paraganglioma
	No significant flow voids	-	-	-
Meningioma	Homogeneous intense enhancement	Slowly progressive CN deficits	Hyperostosis common (25%)	Dural tail sign: bone hyperostosis vs. erosion
	Dural tail sign (70%)	Multiple lower CNs affected	Sclerotic bone reaction	No prominent vascularity
	Isointense on T1 and T2	Older age (50-70 years)	Calcification (20-25%)	Homogeneous enhancement
	Calcification may cause T2 hypointensity	F:M = 3:1	Broad dural attachment	Extra-axial with CSF cleft
	No flow voids	May have hyperostosis	Bone thickening	-
Metastasis	Variable enhancement	Rapid progression (weeks-months)	Aggressive bone destruction	Rapid growth, known primary cancer
	Irregular margins	Known primary malignancy	Lytic lesions	Aggressive bone destruction, perilesional edema
	Perilesional edema common	Multiple CN involvement Constitutional symptoms Age: Variable (usually >50)	Cortical breakthrough	Often multiple sites
	Multiple lesions often	-	Soft tissue mass	-
	Restricted diffusion possible	-	Loss of bone margins	-
Chordoma	Heterogeneous enhancement	Midline destructive lesion, progressive CN deficits	Midline clival/sacral extensive bone destruction	Midline location (clivus), physaliferous cells on histology
	T1: Hypointense	Occipital/neck pain	Calcification (50%) soft tissue mass	Very high T2 signal
	T2: Markedly hyperintense	Age: 50-70 years	Geographic bone lysis	Extensive bone destruction is rare at the jugular foramen
	Honeycomb appearance	M: F = 2: 1	-	-
	Septations common	-	-	-
Lymphoma	Homogeneous enhancement	Rapid progression	Permeative pattern	Restricted diffusion
	Restricted diffusion (high cellularity)	Multiple CN involvement B symptoms (fever, weight loss)	Minimal bone destruction initially	Rapid progression
	T1: Iso- to hypointense	May have systemic lymphoma	Infiltrative appearance	Responds to steroids/chemo
	T2: Iso- to hypointense	Age: Variable	May preserve bone architecture early	Systemic involvement
	No necrosis typically	-	-	Homogeneous T2 signal
Inflammatory (sarcoidosis/IgG4-RD)	Diffuse pachymeningeal enhancement	Multiple CN palsies common	Minimal bone changes	Steroid-responsive
	T1: Isointense	Systemic manifestations	No destructive lesion	Systemic disease
	T2: Variable	Elevated inflammatory markers	Soft tissue thickening	Elevated ACE/IgG4
	May mimic meningioma	Responds to steroids	Dural enhancement, preserved bone margins	Leptomeningeal enhancement
	Leptomeningeal involvement	Younger age possible	-	No mass effect typically
Skull base osteomyelitis	Marrow replacement (T1 hypointense)	Severe otalgia	Bone erosion and sclerosis	Clinical context (DM, otitis)
	T2/STIR hyperintense	Diabetes or immunocompromise	Periosteal reaction	Fever and elevated CRP/ESR
	Enhancement of bone and soft tissue	Fevers, elevated inflammatory markers	Sequestrum may be present	Bone marrow edema
	May have abscess formation	Multiple CN palsies (Collet-Sicard)	Air-fluid levels in the mastoid	Responds to antibiotics
	-	External otitis often	Cortical destruction	Adjacent soft tissue inflammation

The anatomical correlation between the tumor's location and the patient's focal neurological deficit favored a paraganglioma with selective mechanical or ischemic compromise of CN XII [17].

The imaging findings directly support the proposed mechanism of selective compression of CN XII. MRI demonstrated a 1.7 × 2.4 × 8.4 cm tumor centered in the jugular foramen with medial extension bringing its margin to within 4-5 mm of the hypoglossal canal. Importantly, CT confirmed that while the jugular foramen showed approximately 40% circumferential erosion, the hypoglossal canal itself remained intact with no bony destruction. This anatomical configuration - tumor proximity without direct canal invasion - suggests extrinsic compression of CN XII at its extracanalicular cisternal segment or at the canal exit, rather than intracanalicular involvement. The sparing of CN IX-XI, despite their passage directly through the tumor-occupied jugular foramen, likely reflects the tumor's eccentric medial growth pattern rather than central expansion within the foramen itself. This spatial selectivity explains the isolated involvement of CN XII without the typical Collet-Sicard syndrome.

Management of skull base paragangliomas depends on patient factors, tumor size, and anatomical extension. While surgical resection remains the standard for select patients, it carries substantial morbidity due to the complexity of the region and potential for cranial nerve injury [18]. In our case, the lesion was slow-growing but functionally active, as demonstrated by elevated plasma catecholamine levels. It was associated with an isolated cranial nerve deficit and symptoms of autonomic hyperactivity (sustained hypertension, palpitations, and diaphoresis). Given these factors and the proximity to critical neurovascular structures, a multidisciplinary team recommended non-surgical intervention. The patient was offered intensity-modulated radiation therapy (IMRT), which was subsequently administered with curative intent, aiming to achieve long-term tumor control while preserving neurological function [19].

Recent studies have demonstrated high local control rates and low complication rates with radiosurgery for similar skull base tumors, further supporting the safety of watchful waiting in selected cases [20]. Given the tumor's functional status (elevated metanephrines), IMRT (fractionated radiation) was recommended as the primary treatment approach. This decision was based on: (a) slow growth pattern typical of paragangliomas, (b) isolated but stable neurological deficit, (c) biochemically active tumor requiring intervention to prevent potential future catecholamine-related complications, and (d) the patient's preference to avoid surgical morbidity. The functional status has important management implications: (a) annual monitoring of plasma metanephrines is required, (b) alpha-blockade would be necessary prior to any surgical intervention, (c) genetic counseling for SDH mutation testing is recommended, and (d) surveillance for multifocal disease is warranted.

Stereotactic radiosurgery with a tumor margin dose of 12-14 Gy is planned, with expected local control rates >90% based on the literature. Following IMRT, the patient will undergo clinical evaluation every three months for the first year, with MRI at six-month intervals, and annual plasma metanephrine monitoring. Table 4 summarizes the workup of this case.

**Table 4:** Diagnostic summary.

Category	Findings
Clinical features	Isolated left cranial nerve XII palsy with no cranial nerve IX-XI involvement; no autonomic symptoms despite elevated metanephrines
Magnetic resonance imaging characteristics	1.74 × 2.4 × 8.4 cm jugular foramen mass with intense enhancement and a characteristic “salt-and-pepper” appearance due to flow voids; proximity to, but no invasion of, the hypoglossal canal
Computed tomography findings	Permeative erosion of the jugular foramen involving approximately 40% of the circumference; hypoglossal canal structurally intact
Laboratory findings	Elevated free normetanephrines (292 pg/mL; reference <148 pg/mL); elevated free metanephrines (63 pg/mL; reference <57 pg/mL); elevated total metanephrines (355 pg/mL; reference <205 pg/mL)
Final diagnosis	Functional jugular paraganglioma with atypical isolated cranial nerve XII involvement and no systemic catecholamine symptoms

## Conclusions

Jugular paraganglioma should be considered in the differential diagnosis of progressive isolated hypoglossal nerve palsy. While these tumors typically affect multiple lower CNs, selective extension into the hypoglossal canal without involvement of adjacent structures is possible. Advanced imaging and neuroanatomical correlation are crucial for accurate diagnosis. Management must be individualized and guided by tumor location, patient symptoms, and potential treatment risks. Multidisciplinary evaluation remains essential to optimize outcomes in rare presentations of cranial neuropathy.

Clinicians should maintain high suspicion for skull base paraganglioma in patients presenting with isolated progressive cranial neuropathy without an alternative explanation, unilateral tongue deviation with fasciculations and atrophy, absence of upper motor neuron signs, and particularly when combined with pulsatile tinnitus or hearing loss, even if mild.
